# Asexual Reproduction Can Account for the High Diversity and Prevalence of Rare Taxa Observed in Microbial Communities

**DOI:** 10.1128/AEM.01099-19

**Published:** 2019-07-18

**Authors:** Cristina M. Herren

**Affiliations:** a Harvard Data Science Initiative, Harvard University, Cambridge, Massachusetts, USA; b Department of Biostatistics, Harvard T. H. Chan School of Public Health, Boston, Massachusetts, USA; c Department of Biomedical Informatics, Harvard Medical School, Boston, Massachusetts, USA; Rutgers, The State University of New Jersey

**Keywords:** Allee effects, asexual reproduction, emergent properties, microbial communities, population dynamics, stochastic model

## Abstract

Recent studies evaluating the community structures of microorganisms and macroorganisms have found greater diversity and rarity within micro-scale communities, compared to macro-scale communities. However, reproductive method has been a confounding factor in these comparisons; the microbes considered generally reproduce asexually, while the macroorganisms considered generally reproduce sexually. Sexual reproduction imposes the constraint of mate finding, which can have significant demographic consequences by depressing birth rates at low population sizes. First, I construct an island biogeography model to study the organization of ecological communities under neutral stochastic processes. Then, I examine theoretically how the effects of mate finding in sexual populations translate to the emergent community properties of diversity, rarity, and dominance (size of the largest population). In mate-limited sexual populations, the decreased growth rates at low population densities translate to a higher extinction rate; this increased extinction rate had a disproportionately strong effect on taxa with low population densities. Thus, mate limitation decreased diversity, primarily by excluding small populations from communities. However, the most abundant taxa were minimally affected by mate limitation. Therefore, mate limitation affected the diversity and rarity of taxa in communities but did not alter the dominance of the largest population. The observed shifts in community structure mirror recent empirical studies of micro-scale versus macro-scale communities, which have shown that microbial communities have greater diversity and rarity than macrobial communities but are not different in dominance. Thus, reproductive method may contribute to observed differences in emergent properties between communities at these two scales.

**IMPORTANCE** There have been numerous recent efforts to integrate microbes into broad-scale ecological theories. Microbial communities are often structurally distinct from macrobial communities, but it is unclear whether these differences are real or whether they are due to the different methodologies used to study communities at these two scales. One major difference between macroorganisms and microorganisms is that microbes are much more likely to reproduce asexually. Sexually reproducing taxa have diminished growth rates at low population size, because they must encounter another member of their species before reproducing. This study shows that communities of asexually reproducing taxa are expected to be more diverse, because taxa persist longer. Furthermore, asexually reproducing taxa can exist at much lower densities than sexually reproducing taxa. Thus, asexual reproduction by microbes can account for two major differences between microbial and macrobial communities, namely, greater diversity and greater prevalence of rare taxa for microbes.

## Abstract (precis)

There have been numerous recent efforts to integrate microbes into broad-scale ecological theories. Microbial communities are often structurally distinct from macrobial communities, but it is unclear whether these differences are real or whether they are due to the different methodologies used to study communities at these two scales. One major difference between macroorganisms and microorganisms is that microbes are much more likely to reproduce asexually. Sexually reproducing taxa have diminished growth rates at low population size, because they must encounter another member of their species before reproducing. This study shows that communities of asexually reproducing taxa are expected to be more diverse, because taxa persist longer. Furthermore, asexually reproducing taxa can exist at much lower densities than sexually reproducing taxa. Thus, asexual reproduction by microbes can account for two major differences between microbial and macrobial communities, namely, greater diversity and greater prevalence of rare taxa for microbes.

## INTRODUCTION

Ecologists have historically been fascinated by the diversity of microbial communities (1), and several recent studies have indeed demonstrated differences in community structure between microbes and larger “macroorganisms” (2–6). Generally, microbial communities have greater diversity, resulting in part from the large number of rare taxa (7). Other properties, however, such as abundance of the most dominant taxon, are indistinguishable between communities on the two different scales (4). Despite increasing data on which to base these comparisons, the mechanisms generating these patterns of population distributions within and among communities are poorly understood (8). One prominent additional difference between many of the microbial populations and macroorganism populations in prior comparative studies is reproductive method; the microbial populations considered (bacteria, archaea, and most phytoplankton) reproduce asexually, while most macroorganism populations studied have sexual reproduction. In empirical studies, however, it is difficult to tease apart the multiple characteristics that are confounded between micro- and macro-scale communities (such as generation time, per-capita growth rates, and body size) to determine the underlying causes of the differing community organizations. For this reason, mathematical models are useful to separate the effects of confounded traits, because a single trait can be evaluated in isolation. Here, I examine theoretically whether reproductive method can contribute to observed differences in community structure between asexually reproducing microorganisms and sexually reproducing macroorganisms.

Individuals in sexually reproducing populations must encounter a mate before reproducing, whereas asexual individuals do not have this constraint. Mate finding and its effects on population dynamics have been extensively studied in the theoretical literature (beginning with Volterra in 1938 [9]), in part because it is one mechanism that causes Allee effects (reviewed in reference 10). An Allee effect is defined as positive density dependence within a population, meaning that individual-level growth rates increase as population density increases (11). When an Allee effect is present, the benefit of encountering another individual from the population outweighs negative interactions, such as competition, and individuals become more reproductively successful as density increases (12). In populations with sexual reproduction, sparse populations are slow growing due to the inability to find a mate. Mate encounters become more frequent as the population grows, such that per-capita fitness increases as density increases. Thus, the effects of mate finding on population growth are prominent when populations are small but decrease when populations are large and mates are no longer limiting (13).

Many previous theoretical models have considered mate finding and Allee effects by using deterministic models, such as differential or difference equations, to describe the population growth rate (11, 13, 14). Strong reductions in birth rates due to mate limitation can cause population declines at low abundance, effectively setting a “critical density” below which the population becomes extinct (15). When the population size is greater than the critical density, the population continues to grow until it reaches a stable equilibrium at its carrying capacity (16). However, a major drawback of deterministic models is the inability to consider the time to extinction for populations with a positive stable equilibrium; with deterministic equations, any population with a stable positive abundance will persist indefinitely. This result conflicts with the empirical observation that smaller populations are more vulnerable to extinction (17).

Stochastic models are promising for studying the demographic consequences of mate finding because they allow for extinction in populations that would, in the absence of stochasticity, reach a positive carrying capacity (18). In contrast to deterministic models that converge to equilibrium abundances, populations in stochastic models fluctuate through time on the basis of birth and death rate probabilities (19). Thus, the birth rates when populations sizes are small should be strongly related to extinction risk, as these birth rates govern the probability that the population size will reach zero. Several forms of stochastic populations models have been used to study populations with Allee effects, often with discrete time models (20–22). Those studies have concluded that diminished growth rates at low population densities can substantially decrease the expected time to extinction (22, 23). However, it is computationally difficult to model multiple interdependent populations or populations with overlapping generations in discrete time models (24), which is often a prohibitive barrier to such studies.

Here I compare population and community dynamics between communities in which populations must find mates before reproducing and communities in which populations have no mate limitation (a case equivalent to asexual reproduction). I use stochastic models to evaluate demographic consequences of mate finding. First, I use continuous-time Markov chain (CTMC) models, which implement births and deaths in individual “reactions,” to study how mate limitation alters the time to extinction for single populations. These models use a computationally efficient simulation algorithm, which allows for simulation of multiple coexisting populations. Such models have been extensively used for simulating chemical reaction networks (25) but also can be used for modeling population dynamics (26). One substantial benefit of reaction-based models (such as CTMC models), in comparison to models introducing white noise into model dynamics, is that the stochasticity is biologically interpretable as variation in the time between births and deaths of individual organisms. After obtaining the mean times to extinction (MTEs) from these models, I use the island biogeography framework to evaluate how varying extinction times translate to changes in community diversity. The island biogeography framework posits that the expected long-term community diversity can be calculated by identifying the number of taxa when immigration and extinction rates are equal (27). In these models, I assume identical immigration rates for the various communities but extinction rates are influenced by mate limitation. Finally, I simulate communities consisting of populations with differing growth rates to evaluate how the consequences of mate limitation scale to heterogeneous communities. I show that the constraint of mate search decreases diversity, primarily by excluding rare taxa, whereas dominance (defined as the size of the largest population [2]) is unaffected.

## RESULTS

### Single-population dynamics.

**FIG 1:**
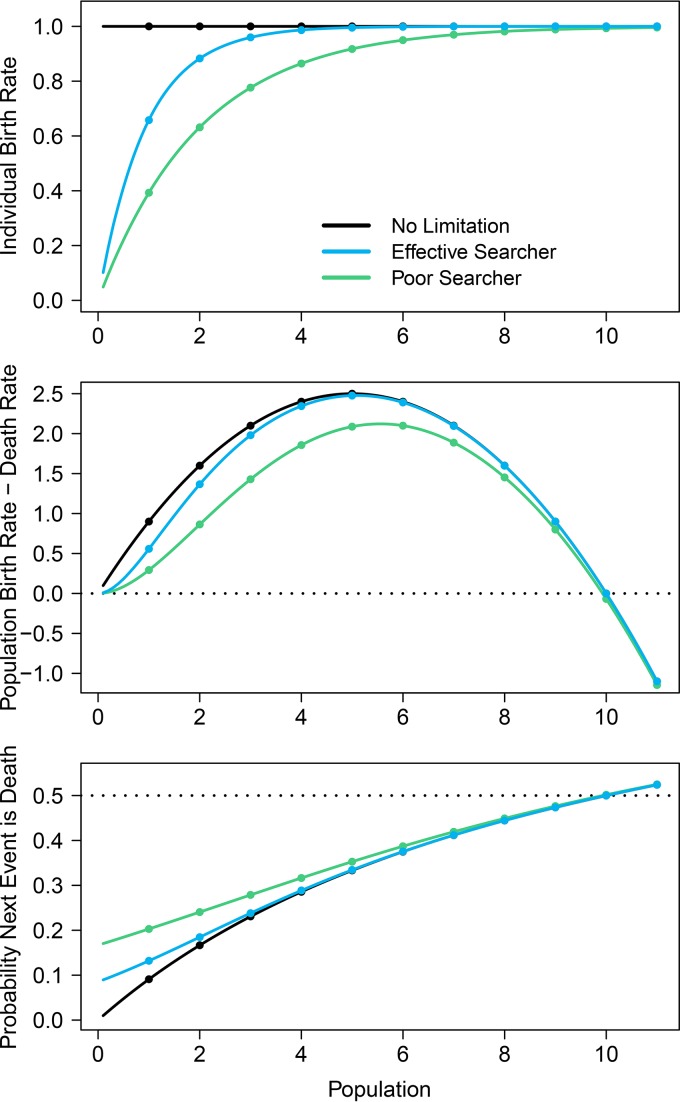
Mate limitation decreases the individual-level birth rate at low population density (top), which influences both the population-level growth rate (middle) and the probability that the next event in the model will be a death (bottom). Effects of mate limitation on population growth become negligible as population sizes increase, as indicated by the convergence of the three scenarios at larger populations. The effect of mate limitation is the difference between the aseuxally reproducing populations (black lines) and the sexually reproducing populations (blue and green lines). Population growth rates are suppressed more strongly for poor mate searchers (green lines) than for effective mate searchers (blue lines). The dotted line in the bottom panel indicates a probability of 0.5, where a birth and a death are equally likely.

First, I compared the times to extinction for nonlimited populations (i.e. asexual reproducers) and for sexual populations in which mates must be encountered. For sexual populations, I evaluated two combinations of search radius and search speed. One scenario indicates a poor searcher with low search radius and speed (*R* = 0.62 and *V* = 0.62), and the other indicates a more effective searcher with greater search radius and speed (*R* = 0.8 and *V* = 0.8). I chose parameter values that would yield equivalent long-term population dynamics if these populations were modeled deterministically; all three scenarios have nearly identical population densities when the birth rate equals the death rate, indicating equal carrying capacities in the absence of stochasticity (Fig. 1, middle). The per-capita birth rate was much higher in small populations for the asexually reproducing populations than for the sexually reproducing populations (Fig. 1, top). However, the birth rate in the sexual populations increased as individuals became more effective at finding mates. Multiplying individual birth and death rates by population size yields population-level birth and death rates; whichever rate is larger indicates which event is more likely to occur next (Fig. 1, middle). Effects of mate limitation are prominent in small populations but negligible as population size increases. With CTMC models, it is also possible to calculate the probability that the next event in the model will be a birth or a death. In the simulated populations with asexual reproduction, it is highly unlikely that a death will occur when the population size is small. This probability of population decline in small populations is increased when mate limitation is present (Fig. 1, bottom).

**FIG 2:**
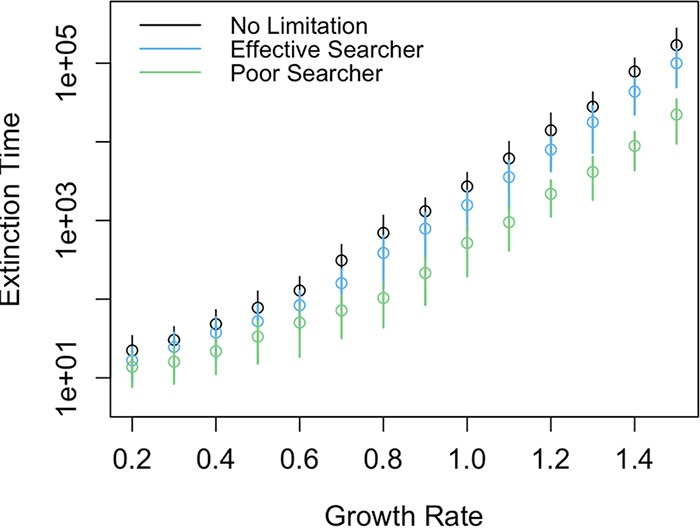
Populations were simulated with different growth rates and different degrees of mate limitation; populations had either no limitation (black), weak limitation due to effective searching (blue), or strong limitation due to poor searching (green). The MTEs for all three types of populations increased approximately log-linearly as population growth rate increased. Populations with no mate limitation had the greatest MTE for any growth rate, and poor searchers had the most rapid extinction. Points and associated lines represent the mean and standard deviation for 100 populations simulated with the same parameters.

I recorded the time to extinction for 100 simulated populations parameterized with the three mate search scenarios shown in Fig. 1 and with varying intrinsic growth rates (*b*). All populations had equivalent death rates. I used initial population sizes of 2, as this is the population size of newly colonizing taxa in the immigration simulation models. Typical model behavior showed the populations increasing in size and then fluctuating around the population size where the birth rate and the death rate were equal. Extinction occurred when population fluctuations were sufficiently large to drive the population to zero. Asexual populations persisted longest among the three scenarios across all values for the intrinsic growth rate. Across the three mate limitation scenarios, the time to extinction increased approximately log-linearly with increasing intrinsic birth rate (Fig. 2). Averaging over 1,000 populations with a growth rate of 1.0, the MTE for asexual populations was 2,664. In mate-limited populations, the time to extinction for effective searchers averaged 1,557, while the MTE for poor searchers was 477.

### Evaluating diversity with island biogeography theory.

**FIG 3:**
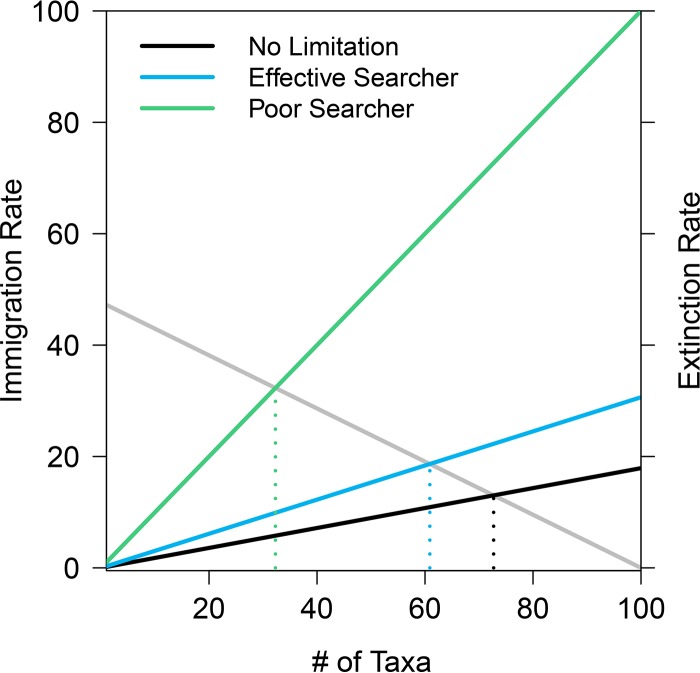
When assuming the same immigration function (gray line; left axis), mate limitation affects expected diversity by changing the time to population extinction. A decreased time to extinction results in a greater slope for the community extinction rate (black, blue, and green lines; right axis). Expected diversity can be found by calculating the number of taxa where the immigration rate and the extinction rate intersect (indicated by dotted lines). Communities are most diverse when there is no mate limitation (black lines). When populations are mate limited but individuals are effective at finding mates, there is a small decrease in expected diversity (blue lines). When individuals are poor searchers, there is a dramatic decline in diversity due to more rapid extinction (green lines). The time scale on which the immigration and extinction rates are shown here is the MTE of the shortest-lived populations (poor searcher populations). Using a different time scale alters the *y* axes but does not change where the lines intersect.

Assuming that a community consisted of populations with identical birth and death rates, I calculated the estimated long-term diversity for the three birth rate scenarios from the associated extinction rates (Fig. 2). The extinction rate for a single population is 1/MTE (with units of taxa per unit time), meaning that the extinction rate for a community of *m* independent taxa is *m*/MTE. I used the same rate of immigration in each scenario. The immigration rate was a linearly decreasing function of current diversity and reached 0 when 100 taxa were present (Fig. 3). It is possible to calculate approximate long-term diversity (number of taxa) by solving for the diversity level when the immigration rate equals the extinction rate (equation 1). For the expected diversity calculations and associated simulations, I used an immigration constant *i* = 0.001 immigrating taxa per unit time, which determines the slope and intercept of the immigration function. However, the stochastic nature of the simulations means that these calculations will be inexact, because the populations never reach equilibrium.

(1) $$\text{Expected diversity}=\frac{\text{no. of colonizing taxa}\cdot i}{\frac{1}{\text{MTE}}+i}$$

Equation 1 shows that the long-term diversity is a function of MTE. As MTE approaches infinity, the expected diversity approaches the diversity level where immigration is zero (in this case, 100). Conversely, as MTE approaches zero, the expected diversity also approaches zero. I evaluated the accuracy of this approximation by using explicit simulations of simultaneously coexisting populations using the same parameters. The two estimates of diversity were within 1 unit (taxon). Approximations using equation 1 yielded expected long-term diversities of 72.6, 60.9, and 32.3 for the scenarios of no limitation, effective searching, and poor searching, respectively, while simulations yielded long-term average diversities of 73.1, 61.8, and 32.9, respectively.

### Comparing communities containing identical versus heterogeneous taxa.

**FIG 4:**
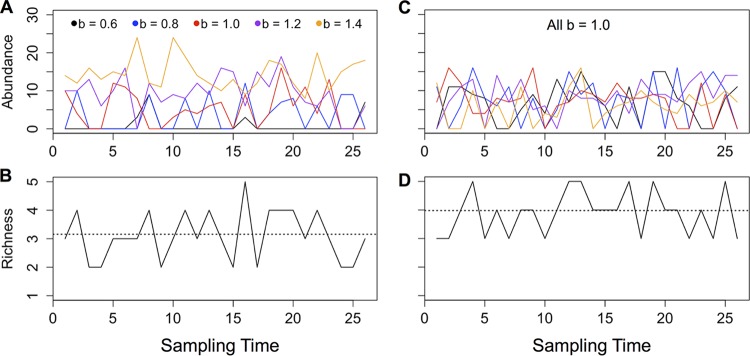
(A and C) Abundances of populations over time in simulated communities containing five taxa. Taxa have either heterogeneous growth rates (A) or identical growth rates (C). (B and D) Diversity (number of taxa present) of the communities over time, with dotted lines indicating mean diversity.

**FIG 5:**
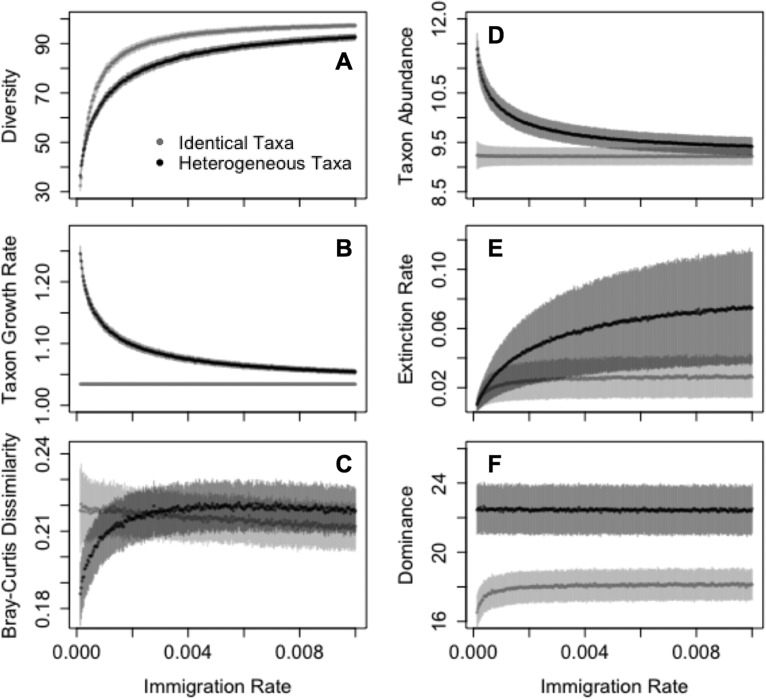
Most emergent properties of simulated communities change in response to changes in the immigration rate used in the model. Communities containing heterogeneous taxa (black) become more diverse (A) and slightly more compositionally stable (C) as immigration increases, but the average growth rate (B) and population size (D) decline and extinctions become more common (E). In populations of identical taxa (gray), diversity increases more rapidly as immigration increases (A), although the mean growth rate (B) and mean abundance (D) are not affected; additionally, communities become slightly less compositionally stable (C), and the extinction rate saturates as a function of immigration rate (E). For both types of communities, dominance (F) is minimally affected by immigration rate except at very low immigration rates, where few taxa are present. The graphs show the mean values (solid points) plus or minus one half standard deviation (shaded bars).

Although the previous diversity approximations were accurate under the assumption that taxa were identical, empirical communities contain taxa with a wide range of population sizes. Therefore, I explored how introducing variability in growth rate (and thus expected population size) would influence the assembly of communities containing asexually reproducing taxa (Fig. 4). I simulated communities containing either taxa with log-normally distributed growth rates or taxa with growth rates equal to the mean of the log-normally distributed growth rates. I recorded the resulting diversity, population size, mean growth rate of extant taxa, dominance, compositional change (Bray-Curtis dissimilarity), and extinction rate (calculated as 1 divided by the time between taxon extinctions) of the communities. Communities containing heterogeneous taxa showed differences from communities containing identical taxa across all of these emergent properties (Fig. 5). For both types of communities, increasing the immigration rate increased the mean diversity, with diversity saturating at high immigration rates. However, communities with heterogeneous taxa were generally lower in diversity as a result of the high extinction rates of the low-abundance taxa. The demographics of heterogeneous communities also shifted in response to the immigration rate; at low immigration rates, the community of heterogeneous taxa was composed mainly of taxa with relatively high intrinsic growth rates and thus long times to extinction. Therefore, changing the immigration rate also effectively altered the community-level extinction rate. Additionally, heterogeneous communities were more compositionally stable at low immigration rates, whereas communities of identical taxa were slightly more stable at higher immigration rates. Dominance was relatively constant across immigration rates for both types of communities, although populations reached greater maximum sizes in the heterogeneous communities due to the presence of taxa with higher growth rates.

### Simulation of community structure with heterogeneous taxa.

**FIG 6:**
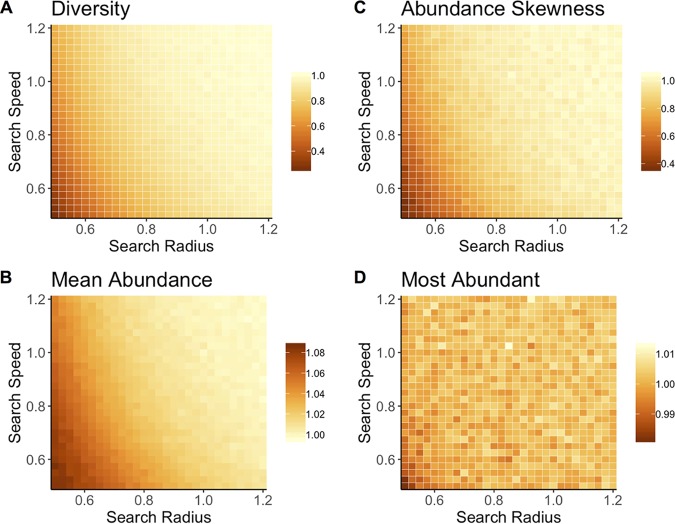
Heatmaps show average diversity (A), average abundance (B), skewness of population abundances (C), and dominant population size (D) from simulations of mate-limited communities in which populations have varying search radius (units of length) and search speed (units of length per time). The mate search equations assume that individuals in the population are distributed randomly at a density of *N* per unit volume (length^3^). Cells within each heatmap show the results for communities consisting of populations with the given search radius and search speed. Cell values are scaled to results from communities without mate limitation. Thus, a value of 1 indicates results equivalent to those of nonlimited communities. The diversity (A), mean abundance size (B), and population size skewness (C) within communities containing mate-limited populations change in response to mate limitation, with stronger mate limitation corresponding to decreased diversity, larger average population size, and lower skewness. When the search radius and search speed are large, the probability of finding a mate approaches 1, and results for mate-limited and nonlimited communities converge. The size of the most abundant population (D) is minimally affected by mate limitation.

Finally, I compared diversity, mean population size, and dominance (population size of the most abundant taxon) of communities containing mate-limited taxa and those with nonlimited taxa. When sexually reproducing taxa were highly effective searchers due to high search radius and/or speed values, mate limitation had little effect on the effective birth rates of those taxa. Then, diversity and population size converged with results from communities containing asexual populations (Fig. 6). However, the abundance of the most dominant population was minimally affected by search efficacy or reproductive method (Fig. 6). Cells in Fig. 6 are scaled according to the values from the no-limitation simulations (a value of 1 indicates equivalent results).

Communities containing the poorest mate searchers experienced the greatest declines in diversity, in comparison with the communities with asexual populations. The communities with the strongest mate search limitation (*R* = 0.55 and *V* = 0.55) had a mean diversity of 27.4 taxa; conversely, communities in which mates were not limiting had a mean diversity of 67.2 taxa. Similarly, mean population size was 11.0 for the most mate-limited communities but 10.2 for nonlimited communities. Another measurement of species abundance distribution, the skewness of population abundances, showed a similar result; higher skewness indicates a greater proportion of low-abundance taxa, and skewness was near its maximum in communities with asexual taxa. Average skewness in the distribution of population sizes was 0.269 for communities with the greatest mate limitation and 0.565 for nonlimited communities. However, dominance was not consistently related to mate limitation. The size of the dominant population in communities with mate-limited populations could be higher or lower than the dominant population in nonlimited communities. The smallest values of *R* and *V* represent the point at which mate searching could become limiting for the largest populations in the communities; in this case, mean dominance declined by approximately 1%. In mate-searching populations, the same degree of limitation could be generated with different combinations of search radius and search speed. Any combination of *R* and *V* that produces a constant value of *VR^2^* yields an equivalent probability of encountering a mate (see equation 4).

## DISCUSSION

This study presents a stochastic framework for studying the assembly of microbial communities and further shows that mate limitation influences emergent community properties, including diversity and average population size. Mate limitation strongly suppresses birth rates when populations are small (Fig. 1), leading to a higher probability that sexually reproducing populations will decline when rare. These discrepancies in birth rate lead to shorter times to extinction in taxa that must find a mate, versus those that reproduce asexually (Fig. 2). This effect is particularly strong when populations are introduced at low density, which is a plausible scenario when considering newly established populations. In stochastic simulations, communities consisting of asexual taxa maintained greater diversity due to a longer expected persistence time for each population (Fig. 3). In the case in which immigration is a linear function of current diversity, expected diversity increases as MTE increases (equation 1). In communities containing heterogeneous taxa, the rapid turnover of small populations drove the changes in the emergent properties of diversity and mean population size (Fig. 5). When mate limitation was added to these simulations, differences in diversity and rarity were amplified, because mate limitation had especially strong negative effects on taxa with already-low growth rates (Fig. 6). Thus, mate limitation decreased the number of coexisting taxa, primarily by excluding low-abundance taxa. Mate limitation had minimal consequences in larger populations, however, and thus the population size of the most abundant taxon was not related to reproductive method or mate search efficacy.

The degree of mate limitation is a function of search ability, which is determined by search radius and search speed. As either search variable (radius or speed) increases, the probability of finding a mate approaches 1, indicating no limitation for the population birth rate. In this case, simulation results for sexual populations with mate finding converge with those for populations without limitation. This is also evident when looking at per-capita and population growth curves (Fig. 1). Birth rates asymptotically reach the no-limitation case as mate searching becomes more effective. Furthermore, this study highlights the utility of stochastic models for studying community structures. If the same populations considered in Fig. 1 were modeled with deterministic equations, then the populations would reach identical carrying capacities, whereas these stochastic models showed pronounced differences. This study broadly concurs with prior models showing that Allee effects increase extinction rates (28–30), and it further demonstrates that these population-level effects alter emergent community properties.

Results from these models mirror empirical findings that microbial populations (with asexual reproduction) tend to be high in diversity and rarity, although not distinct from other communities in the dominance of abundant taxa (2). In these simulated communities, eliminating the constraint of mate finding translated to greater diversity with a higher frequency of low-abundance taxa, while the population size of the most abundant taxon was unaffected (Fig. 6). Thus, allowing for mate finding generates a parsimonious explanation for the community-level patterns observed in comparisons of micro- and macro-scale ecological communities. However, a heightened rate of immigration in microbial communities could be an alternative explanation, as increasing immigration also led to greater diversity and smaller average population size (Fig. 4). Using empirical data to probe the hypothesis that mate limitation constrains diversity and rarity illustrates the plausibility of the mate limitation explanation. For example, one study used very deep 16S amplicon sequencing to evaluate whether marine bacterial populations that appeared to be present only seasonally were instead consistently present at abundances below the usual detection limit (31). At a depth of approximately 11 million sequences, 48% of sequences appeared only once (31). Similarly, another study used deep sequencing of human gut samples to generate rarefaction curves illustrating how many taxa were observed in response to sequencing depth. New taxa continued to be identified after 1 million sequences were recovered (32). Even if many of the observed rare taxa are the products of sequencing errors, these findings suggest persistence of extremely low-abundance taxa (fewer than 1 individual per million). For sexually reproducing populations, these relative abundances could be prohibitively low for individuals to find mates within a lifetime. Finally, asexual reproduction in microbes makes it possible for single individuals to establish populations in new environments. Given the demonstrated plausibility of immigration from microbial seed banks (31, 33), the growth rate of small populations is especially relevant for the persistence of microbial taxa.

Diversity is a common variable of interest in ecological studies, although there is ongoing debate regarding how diversity is related to community function (8). In the models studied here, diversity is a by-product of population demographics, including birth rate and mate search ability. More generally, these models show that diversity, as well as other emergent properties, can be affected by neutral and stochastic processes. For example, among communities containing heterogeneous taxa (Fig. 5), more diverse communities were slightly less stable in composition, because both diversity and compositional change could be influenced by average population growth rates. Subsequent empirical studies using diversity as an outcome variable might also collect information about immigration and extinction rates, to determine whether diversity reflects these processes. For example, surveys of human-associated microbial communities have found variation in diversity across body sites (34, 35). Gut and oral bacterial communities are especially diverse (35), but these habitats could conceivably have higher immigration rates than other body sites due to daily introduction of bacteria within food (36). Similarly, a recent study found little evidence that fungi could persist within the healthy human gut but still identified hundreds of fungal taxa in stool samples (37). The high diversity of fungi in the human gut, despite their inability to colonize this habitat, was attributed to persistent immigration of fungi on ingested foods (37). These studies, coupled with the modeling results presented here, demonstrate how diversity could change independently of community function.

The simulations in which taxa have identical birth rates (Fig. 5) represent a neutral model of community assembly and show what emergent properties are expected from only stochastic processes. Hubbell’s original neutral theory of biodiversity (38) has been able to provide a good fit to macro-scale data sets by assuming that individuals in communities are randomly replaced either by a new member of an existing species or by an immigrating species. However, recent tests of neutral theory in human microbiome communities found that neutral models generally fit such data poorly (39, 40). As the volume of publicly available genomic data has grown, microbial biogeography and biodiversity studies have increasingly used global-scale microbial data sets to empirically describe microbial diversity (41–44). Those surveys have found that a small number of bacterial taxa are widely distributed and often abundant (42–44); thus, the distribution of dominant taxa diverges from the neutral expectation, because a small subset of taxa are disproportionately dominant across multiple communities. In contrast to the similarity of globally distributed microbial communities in terms of their abundant taxa and community structure, rare taxa are more localized to communities (7). However, rare taxa can be disproportionately important in community assembly, compositional change, and function (7, 45–47). Thus, the enrichment of rare taxa in microbial communities (compared to distributions from theoretical models and studies of macro-scale communities) might imply that microbial communities also have different expected baselines for compositional and functional stability.

In addition to the population dynamics of asexual versus sexual populations, there are other factors that likely contribute to observed differences in the structure of microbial communities. First, micro- and macro-scale communities are studied with different empirical methods, which raises the question of whether the differences in emergent properties could be produced by differential biases in methodology. Specifically, DNA sequence similarity is often used to define microbial taxa, whereas macroorganisms are generally identified using direct observations. The differences in error rates and detection limits between these two methods could also explain the greater diversity and rarity in microbial communities. Several steps in the workflow for generating 16S amplicon data, including variations in sample processing and sequencing errors, can generate observations of artifactual rare taxa (48). Furthermore, macroorganisms are often identified using morphological characteristics, but many more taxa can be differentiated if DNA sequencing methods are used (49). Thus, although methodology is confounded in many empirical studies of the diversity and rarity of micro- and macro-scale communities, the mathematical models that are free of these limitations produce similar patterns, compared with the empirical data. In addition to reproductive method, there are further confounded mechanisms between micro- and macro-scale communities that could contribute to the observed differences in community structure. For example, cross-feeding, chemical interference, and dormancy are common in microbial systems and can increase the expected diversity of communities (50–52); including these mechanisms in ecological models allows the number of coexisting taxa to greatly exceed the number of nutrients in the system, which would otherwise limit coexistence (53). More generally, future models might incorporate the influence of biotic interactions on community assembly, as these interactions are known to shape the persistence and abundance of taxa. Finally, the high level of diversity of microscopic eukaryotes has been recognized for decades and has been attributed in part to their large population sizes (in comparison with larger eukaryotes), the large number of habitats available to them as a result of their small body size, and their ease of dispersal (54, 55). Thus, the observed differences in demographic and biogeographic patterns between micro- and macro-scale communities can result from multiple factors acting in combination, although reproductive method may be particularly important due to its high prevalence across microbial taxa.

## MATERIALS AND METHODS

### Single-population dynamics.

First, I studied the effects of mate limitation on the time to extinction for single populations. I used CTMC models to evaluate the time to extinction, as implemented with the Gillespie algorithm (56). Briefly, these models record births and deaths in a population as events that occur with varying frequency, depending on population size. Births are marked by the addition of a single individual to the population, whereas deaths remove a single individual. The overall rate at which any event (birth or death) occurs is the sum of the birth and death rates. The time until the next event is exponentially distributed with a parameter equal to the summed event rates. Therefore, as event rates increase, waiting time until the next event decreases. After drawing of a random value from the exponential distribution for the time increment, the magnitudes of the instantaneous birth and death rates indicate whether a birth (add one individual) or death (remove one individual) is more likely to occur. Another random number is generated to determine whether a birth or death event transpires. After an individual is added or removed from the population, birth and death rates are updated based on the new population size, and the steps repeat. Extinction occurs at the first time point at which the population equals zero.

Throughout this study, I consider populations that are self-limiting. In deterministic models, self-limiting populations experiencing logistic growth reach a stable carrying capacity determined by the intrinsic birth rate (*b*) and the density-dependent death rate (*d*) (equation 2).

(2) $$\frac{\mathrm{dN}}{\mathrm{dt}}=\mathrm{bN}-dN^{2}$$

In the stochastic model formulation, births and deaths are modeled as discrete events, also referred to as “reactions” (57). A birth reaction occurs when a single individual turns into two identical individuals. The birth rate parameter, *b*, is the per-capita birth rate of the population (with units of individuals per unit time). For example, at a birth rate of 1.2, the expected waiting time between births for a single individual is exponentially distributed with a rate parameter of 1.2; then, the number of births within 1-unit time intervals is Poisson distributed with a rate parameter of 1.2. If individuals reproduce independently, then at a population size of *N*, the rate at which a single individual is added to the population is the per-capita birth rate (*b*) multiplied by population size (*N*) (equation 3).

(3) For event N→N+1, rate=bN

To study the effects of mate limitation, I modified the birth event rate to include mate search. Previous work has yielded an equation governing the encounter rate between one individual and other individuals when randomly distributed organisms are moving in three-dimensional environments (58). The mate encounter rate is dependent on the speed at which individuals move (*V*) and the radius at which they can detect a mate (*R*). Here, I assume that males and females move at the same speed and that there is a 1:1 male/female ratio. Multiplying the intrinsic birth rate (*b*) by the probability that at least 1 mate will be encountered (58) yields the following birth event rate for mate-limited populations (equation 4):

(4) $$\text{For event }N\to N+1\text{, rate}=\mathrm{bN}\cdot \left(1-e^{-2\pi R^{2}VN/3}\right)$$

The death rate functions used for the two cases were identical and were chosen to be analogous to the death rate in equation 2. In this case, the individual-level death reaction occurs when a single individual is removed from the population. The individual-level death rate (*dN*, with units of individuals per unit time) is a linearly increasing function of current population size, which leads to self-limitation in population size. In a population in which deaths of individuals are independent, the rate of any death occurring in the population is the product of the individual-level death rate and the number of individuals (*N*) in the population (equation 5).

(5) $$\text{For event }N\to N-1\text{, rate}=dN^{2}$$

As an illustration of the difference between deterministic and stochastic models, I investigated cases in which long-term population dynamics of the various populations would be equivalent in the deterministic case; both mate-limited and nonlimited populations would have nearly identical carrying capacities if these dynamics were translated to deterministic models. I simulated population trajectories and evaluated the time to extinction using CTMC models. I used the two birth rate expressions (equations 3 and 4) for the scenarios with and without mate limitation and equation 5 for the death rate in all models. Additionally, I simulated populations with different intrinsic birth rates. I recorded the MTE for 100 simulated chains (populations) for each combination of mate searching and intrinsic growth rate.

### Evaluating diversity with island biogeography theory.

The theory of island biogeography formalized the concept that long-term community diversity is governed by the rate at which taxa enter the community (i.e., immigration) and the rate at which taxa leave the community (i.e., extinction). In island biogeography models, the immigration rate and extinction rate of taxa within a community change as a function of the number of taxa currently present in a community (27). Thus, the expected diversity (defined here as the number of coexisting taxa) of the community is identified by finding the number of taxa at which the immigration and extinction rates are equal. To evaluate the accuracy of this analytical approximation for these simulations, I calculated the expected long-term diversity for a community consisting of populations with identical birth and death rates (and thus identical MTEs).

I compared results of the analytical estimates of diversity to simulations of diversity in a stochastic reaction network model (coupled simultaneous CTMC model) explicitly tracking each population. In the stochastic reaction network, the community-level immigration event rate was a function of current diversity. Immigration events were modeled as a population increasing from 0 to a small population size, in this case 2 individuals. The rate of a single taxon colonizing was given by *i* (with units of taxa per unit time); therefore, the rate of a colonization event by any taxon is equal to the individual-taxon-level immigration rate multiplied by the number of possible colonizing taxa that are currently absent from the community (equation 6).

(6) For event 0→2, rate=(no. of colonizing taxa−current diversity)·i

Here I assumed that the maximum number of taxa that could colonize the community was 100. I also tested values of 200 and 500, to ensure that results were robust for different community sizes. I conducted these simulations across different parameters governing mate finding.

### Simulation of community structure with heterogeneous taxa.

To study how demographic consequences of mate finding scale to communities with heterogeneous populations, I generated communities in which intrinsic growth rates (*b*) varied among populations. Using these communities, I evaluated how changes in mate-searching parameters affected the diversity, rarity, and dominance of taxa. The equation governing mate searching assumes that individuals within a population are randomly distributed at a density of *N* taxa per unit volume (e.g., per cubic meter) and move randomly while searching for mates. Taxa within each community experienced the same degree of mate limitation, which was determined by changing the values of *R* (search radius, with units of distance [e.g., meters]) and *V* (search speed, with units of distance per time [e.g., meters per second]) over the range of 0.55 to 1.2. Across these combinations of search radius and search speed, mate finding is limiting for population growth in small populations but is not limiting in large populations (those with 20 or more individuals).

For each combination of search radius and search speed for the mate-limited populations, I simulated a stochastic reaction network in which intrinsic birth parameters (*b*) were randomly drawn from a log-normal distribution for which the mean of the underlying normal distribution was 0 and the standard deviation was 0.25. I used a log-normal distribution of growth rates because a log-normal distribution provides a good fit to the observed abundance distributions of microbial taxa (4). However, I verified that simulation results were qualitatively similar when a normal distribution of birth rates was used. Additionally, populations defined by these growth rates routinely become extinct within computationally tractable time scales. Each simulation used the same pool of 100 taxa as potential colonizing populations. Additionally, populations could recolonize after becoming extinct. The death rate constant (*d*) was fixed at 0.1 for all populations. Again, immigration was a linearly decreasing function of current diversity, where an immigration event was modeled as a change in population size from 0 to 2 (equation 6). After a burn-in period with 10,000,000 events, I recorded instantaneous measurements of diversity, dominance (largest population size), and mean population size every 200,000 events. I chose a sampling interval of 200,000 events because at least 20 to 30 immigration events occurred during this time window, which was much larger than the standard deviation of community diversity; therefore, I reasoned that samples spaced with this interval would be sufficiently uncorrelated to represent long-term model behavior, because communities underwent substantial reorganization between samples. I compared results of simulations in which mates were limiting to results of simulations in which mates were not limiting.

### Data availability.

The R scripts used to generate the main results are provided in the supplemental material.

## Supplementary Material

Supplemental file 1
