# Supplementary material for: Asexual Reproduction Can Account for the High Diversity and Prevalence of Rare Taxa Observed in Microbial Communities
Source: Appl Environ Microbiol. 2019 Jul 18;85(15):e01099-19. doi: 10.1128/AEM.01099-19 (PMC6643231; doi:10.1128/AEM.01099-19)
Supplement: Supplemental file 1 [file AEM.01099-19-s0001.pdf]

```
#Supplementary file for generating Fig. 2
#CMH February 2019

#Making plots to visualize time to extinction by mate limitation
and growth rate

#set vector of population growth rates to evaluate
r.vec <- seq(.2, 1.5, .1)

#Decide how many runs to do
runs <- 100
#set density dependent death rate
d.fix <- .1

par(mfrow = c(1, 1))

#Create vectors for extinction time and initial population size
ext1 <- vector()
growth.rate <- vector()

for(birth.fxn in c("no.lim", "good", "poor")){

for(r in r.vec){

    init.abun <- 2
    t1.abun <- init.abun

    for(run in 1:runs){

    set.seed(888 + run)
    seeds <- runif(100000000, 1, 10000000)

    time <- vector()
    time[1] <- 0
    steps <- 1
    abun.vec <- vector()
    abun.vec[1] <- t1.abun

    if(birth.fxn == "no.lim" ) b1.fxn <- function(inst.abun)
inst.abun * r
    if(birth.fxn == "good")    b1.fxn <- function(inst.abun)
inst.abun * r * (1 - exp(-(pi * .8^2 * (inst.abun/2)  * 4 * .8 )/
3)  )
    if(birth.fxn == "poor")    b1.fxn <- function(inst.abun)
inst.abun * r * (1 - exp(-(pi * .62^2 * (inst.abun/2)  * 4 * .62
)/ 3)  )

    while(abun.vec[steps] > 0 ){
```

```
      #Make sure seeds will restart if exhausted
      seed.pick <- max((run + steps) %% length(seeds), 1)

      b1 <- b1.fxn(abun.vec[steps])
      d1 <- abun.vec[steps] *abun.vec[steps] *d.fix
      set.seed(seeds[seed.pick])
      var1 <- rexp(1, b1 + d1)
      set.seed(-seeds[seed.pick])
      var2 <- runif(1)

       if(var2 < b1/(b1 + d1)){
        abun.vec[steps + 1] <- abun.vec[steps] + 1
      } else {
         abun.vec[steps + 1] <- abun.vec[steps] - 1
      }

    time[steps + 1] <- time[steps] + var1
    steps <- steps + 1

   #if(steps %% 10000 == 0) print(steps)

   }

  #Find what entry to fill
  entry <- (which(r.vec %in% r) - 1)*runs + run
  ext1[entry] <- time[length(time)]
  growth.rate[entry] <- r

  if(run %% 10 == 0) print(run)

}

}
  ext.time.mean <- tapply(ext1, growth.rate, mean)
  ext.time.sd <- tapply(ext1, growth.rate, sd)

  ## Name vectors generated for mean and sd so plot can be
produced afterward
  if(birth.fxn == "no.lim") {
    ext.time.nolim <- ext.time.mean
    ext.sd.nolim <- ext.time.sd
  }
  if(birth.fxn == "good") {
    ext.time.good <- ext.time.mean
    ext.sd.good <- ext.time.sd
  }
  if(birth.fxn == "poor") {
    ext.time.poor <- ext.time.mean
```

```
    ext.sd.poor <- ext.time.sd
  }

  ## Make plot during run
  if(birth.fxn == "no.lim") {
    plot(ext.time.mean ~ names(ext.time.mean), log = "y", ylim =
c(5, max(ext.time.mean + ext.time.sd)), xlab = "Growth Rate",
ylab = "Extinction Time", cex.lab = 1.2, cex.axis = 1.2 )
    segments(x0 = as.numeric(names(ext.time.mean)), x1 =
as.numeric(names(ext.time.mean)), y0 = ext.time.mean -
.5*ext.time.sd, y1 = ext.time.mean + .5*ext.time.sd )
  } else {
    if(birth.fxn == "good") color.plot <- "deepskyblue2" else
color.plot <- "seagreen3"
    points(ext.time.mean ~ names(ext.time.mean),  ylim = c(10,
max(ext.time.mean + ext.time.sd)), col = color.plot )
    segments(x0 = as.numeric(names(ext.time.mean)), x1 =
as.numeric(names(ext.time.mean)), y0 = ext.time.mean -
.5*ext.time.sd, y1 = ext.time.mean + .5*ext.time.sd , col =
color.plot, lwd = 1.5)
    legend("topleft", bty = "n", col = c("black", "deepskyblue2",
"seagreen3"), lwd = 1.3, legend = c("No Limitation", "Effective
Searcher", "Poor Searcher"))
  }

}
```

```
## File to generate CTMC of communities containing asexual taxa


# Create necessary functions
rem.fxn <- function(num, tax){
  if(num %% tax == 0){
    return(tax)
  } else {
    return(num %% tax)
  }
}

rxn.fxn <- function(index, tax){
  if(index %% tax == 0){
    return(index / tax)
  } else {
    return(1 + index / tax)
  }
}

min.non.zero <- function(vec){
  min.pos <- min(vec[vec > 0])
  return(min.pos)
}

#Need a function to calculate skewness of rank distribution
rank.vec <- function(vec){
  new.vec <- rep(rank(-vec, ties.method = "first"), times = vec )
  return(new.vec)
}

skew.f <- function(x){
  if( sum(x > 0) <= 1) {
    return(0)
  } else {
    # xrank <- rep(rank(-x, ties.method = "first"), times = x )
    xrank <- x[x > 0]
    n <- length(xrank)
    sk <- (sum((xrank - mean(xrank))^3)/n)/(sum((xrank -
mean(xrank))^2)/n)^(3/2)
    return(sk)
  }
}

med.non.zero <- function(vec){
  non.zero <- vec[vec > 0]
  medn <- median(non.zero)
  return(medn)
}
```

```
mean.non.zero <- function(vec){
  non.zero <- vec[vec > 0]
  medn <- mean(non.zero)
  return(medn)
}

div.fun <- function(vec){
  npresent <- length(vec[vec > 0])
}

npres.vec <- vector()
steps.vec <- vector()
dom.vec <- vector()
tot.vec <- vector()
ab.mat <- vector()

ntaxa <- 100
dall <- .1
ravg <- 0
rsd <- .25
#radfix <- .4
#speedfix <- .8
immc <- .001

#choose a time to start sampling
min.sample.time <- 10000000
sample.int <- 200000
sample <- 1
num.samples <- 1000 # usually 1000

#Choose time to run model
timemax <- min.sample.time + sample.int * num.samples #should
this be somewhat related to MTE?
stepsmax <- min.sample.time + sample.int * num.samples

#generate reactions
rxns <- c(1, -1, 2)
#create counter for introductions
rx3 <- 0

# generate random growth rates
set.seed(88)
r0vec <- exp(rnorm(ntaxa, ravg, rsd))
r0vec <- abs(r0vec)
#generate empty reaction matrix
rx.mat <- matrix(numeric(0), ntaxa, 3)

#initialize abundances
```

```
set.seed(88)
ab <- rpois(ntaxa, 2)
#initialize time
time.inst <- 0
#initialize steps
steps <- 0

par(mfrow = c(1, 1))
plot(0, 0, xlim = c(0, stepsmax), ylim = c(0, ntaxa), xlab =
"Steps", ylab = "Number Present", col = "white")

# start loop
#while(time.inst < timemax){
while(steps < stepsmax){
npres <- sum(ab > 0)
nabs <- sum(ab == 0)
rx.mat[, 1] <- r0vec * ab
rx.mat[, 2] <- ab^2 * dall
rx.mat[, 3] <- 0
if(nabs > 0) rx.mat[, 3][ab == 0] <- immc

rate.tot <- sum(rx.mat)
var1 <- rexp(1, rate.tot)
var2 <- runif(1)

rx.mat.norm <- rx.mat / rate.tot
index <- max(which(c(0, cumsum(rx.mat.norm)) < var2 ) )

tax.react <- rem.fxn(index, ntaxa)
rxn.number <- rxn.fxn(index, ntaxa)

if(rxn.number == 3){
  # r0vec[tax.react] <- exp(rnorm(1, ravg, rsd)) #this would add
a new taxon with a new growth rate
  set.seed(steps)
  # count how many times immigration events happen to check that
model is working as expected
  rx3 <- rx3 +1
}

ab[tax.react] <- ab[tax.react] + rxns[rxn.number]
time.inst <- time.inst + var1
steps <- steps + 1

if(steps %% 1000000 == 0) {
  points(npres ~ steps, cex = .3)
  print(c(npres, max(ab), mean(ab) ) )
}
```

```
  if(steps > min.sample.time){

  npres.vec[sample] <- npres
  steps.vec[sample] <- steps
  dom.vec[sample] <- max(ab)
  tot.vec[sample] <- sum(ab)
  ab.mat <- rbind(ab.mat, ab)
  min.sample.time <- min.sample.time + sample.int
  sample <- sample + 1
  if(sample %% 5 == 0) print(c(sample))
  }

}



mean.sk <- mean(apply(ab.mat, 1, skew.f))
mean.pres <- mean(npres.vec)
mean.dom <- mean(dom.vec)
mean.abun <- mean( apply(ab.mat, 1, mean.non.zero) )

print(c(mean.sk, mean.pres, mean.dom, mean.abun))
```

```
#!/usr/bin/env Rscript

#Arguments input should be search radius, search speed, and
number of runs

args = commandArgs(trailingOnly=TRUE)


# Create necessary functions
rem.fxn <- function(num, tax){
  if(num %% tax == 0){
    return(tax)
  } else {
    return(num %% tax)
  }
}

rxn.fxn <- function(index, tax){
  if(index %% tax == 0){
    return(index / tax)
  } else {
    return(1 + index / tax)
  }
}

mean.non.zero <- function(vec){
  mean.pos <- mean(vec[vec > 0])
  return(mean.pos)
}

#Need a function to calculate skewness of rank distribution
skew.vec <- function(vec){
  new.vec <- rep(rank(-vec, ties.method = "first"), times = vec )
  skew <- skewness(new.vec)
}

skew.f <- function(x){
  if( sum(x > 0) <= 1) {
    return(0)
  } else {
    # xrank <- rep(rank(-x, ties.method = "first"), times = x )
    xrank <- x[x > 0]
    n <- length(xrank)
    sk <- (sum((xrank - mean(xrank))^3)/n)/(sum((xrank -
mean(xrank))^2)/n)^(3/2)
    return(sk)
  }
}
```

```
div.fun <- function(vec){
  npresent <- length(vec[vec > 0])
}

bc.fun <- function(vec1, vec2){
  bc <- 1 -  ( ( 2 * sum( apply(rbind(vec1, vec2), 2, min) ) ) /
sum(c(vec1, vec2)) )
}

radfix <- as.numeric(args[1])
speedfix <- as.numeric(args[2])
num.samples <- as.numeric(args[3])

npres.vec <- vector()
steps.vec <- vector()
dom.vec <- vector()
tot.vec <- vector()
ab.mat <- vector()
ab.mat.div <- vector()

ntaxa <- 100
dall <- .1
ravg <- 0
rsd <- .25
immc <- .001

#choose a number of steps to start sampling
min.sample.steps <- 100000000
min.sample.fix <- min.sample.steps
min.sample.time <- min.sample.steps
sample.int <- 200000
sample <- 1
#num.samples <- 1000
#Choose a time to start sampling
sample.int.time <- 100

#Choose time to run model
timemax <- min.sample.steps + sample.int * num.samples #should
this be somewhat related to MTE?
stepsmax <- min.sample.steps + sample.int * num.samples

#generate reactions
rxns <- c(1, -1, 2)
#create counter for introductions
rx3 <- 0

# generate random growth rates
set.seed(88)
r0vec <- exp(rnorm(ntaxa, ravg, sd = rsd))
```

```
#r0vec <- abs(r0vec)
#generate empty reaction matrix
rx.mat <- matrix(numeric(0), ntaxa, 3)

#initialize abundances
set.seed(99)
ab <- rpois(ntaxa, 2)
#initialize time
time.inst <- 0
#initialize steps
steps <- 0
#create vector to hold time when samples are recorded
time.vec <- vector()

#par(mfrow = c(1, 1))
#plot(0, 0, xlim = c(0, stepsmax), ylim = c(0, ntaxa), xlab =
"Steps", ylab = "Number Present", col = "white")

# start loop
#while(time.inst < timemax){
while(steps < stepsmax){
  npres <- sum(ab > 0)
  nabs <- sum(ab == 0)
  rx.mat[, 1] <- ab * r0vec * (1 - exp(-(pi * radfix^2 * (ab/2)
* 4 * speedfix )/ 3)  )
  rx.mat[, 2] <- ab^2 * dall
  rx.mat[, 3] <- 0
  if(nabs > 0) rx.mat[, 3][ab == 0] <- immc

  rate.tot <- sum(rx.mat)
  var1 <- rexp(1, rate.tot)
  var2 <- runif(1)

  rx.mat.norm <- rx.mat / rate.tot
  index <- max(which(c(0, cumsum(rx.mat.norm)) < var2 ) )

  tax.react <- rem.fxn(index, ntaxa)
  rxn.number <- rxn.fxn(index, ntaxa)

  if(rxn.number == 3){
    #r0vec[tax.react] <- exp(rnorm(1, ravg, rsd))
    rx3 <- rx3 +1
    #print(rx3)
  }

  ab[tax.react] <- ab[tax.react] + rxns[rxn.number]
  time.inst <- time.inst + var1
  steps <- steps + 1
```

```
  # if(steps %% 1000000 == 0) points(npres ~ steps, cex = .3)

  if(steps > min.sample.steps){

    if(steps > min.sample.fix & steps < ( min.sample.fix + 
sample.int) ) min.sample.time <- time.inst
    npres.vec[sample] <- npres
    steps.vec[sample] <- steps
    dom.vec[sample] <- max(ab)
    tot.vec[sample] <- sum(ab)
    ab.mat <- rbind(ab.mat, ab)
    min.sample.steps <- min.sample.steps + sample.int
    sample <- sample + 1
    if(sample %% 5 == 0) print(sample)
  }

  if(time.inst > min.sample.time){
    ab.mat.div <- rbind(ab.mat.div, ab)
    min.sample.time <- min.sample.time + sample.int.time
    time.vec <- c(time.vec, time.inst)
  }

}



med.sk <- mean(apply(ab.mat, 1, skew.f))
med.below5 <- mean(apply(ab.mat < 5 & ab.mat > 0, 1, sum) )
med.pres <- mean(npres.vec)
med.dom <- mean(dom.vec)
med.dom.prop <- mean(dom.vec / tot.vec)
med.abun <- mean(apply(ab.mat, 1, mean.non.zero) )

# Create figure showing population trajectories, diversity, and 
BC dissimilarity over time
bc.vec <- vector()

for(i in 1:(dim(ab.mat.div)[1] - 1) ){
  bc.vec[i] <- bc.fun(ab.mat.div[i, ], ab.mat.div[i+1, ])
  #bc.vec[i] <- vegdist(rbind(ab.mat.div[i, ], ab.mat.div[i+1, 
]))
}

div <- apply(ab.mat.div, 1, div.fun)

summary.vec <- c(med.pres, med.dom, med.dom.prop, med.abun, 
med.sk, mean(bc.vec), var(npres.vec), radfix, speedfix, 
num.samples, mean(tot.vec), ravg, rsd)
names(summary.vec) <- c("med.pres", "med.dom", "med.dom.prop", 
"med.abun", "med.sk", "mean.bc", "div.var", "radfix", "speedfix",
```

```
"num.samples", "tot.vec", "ravg", "rsd")
write.csv(t(summary.vec) , "ModelOutput.csv")
```

```
#!/usr/bin/perl
#Custom PERL script to dispatch simulations on computing cluster
use Getopt::Long;

$iterations=1000;
$interval=0.025;
GetOptions ('iterations=i'=> \$iterations,
'interval=i'=> \$interval,
'debug' => \$debug);

sub runcommand { #this lets me switch from testing to running
easily!
    if ($debug) {
        print $_[0]."\n";
    } else {
        print $_[0]."\n";
        return system $_[0];
    }
}

$dispatchbase = "sbatch -p short -n 1 -t 12:0:0 --mem=2000 --
wrap=";

$moduleloads = "module load gcc/6.2.0;module load R/3.4.1;module
load python/3.6.0;module load snakemake/3.12.0;";
#$moduleloads = "module load gcc/6.2.0;module load R/3.4.1;"

for ($speed=0.5;$speed<=1.2;$speed+= $interval) {
    for ($rad=0.5;$rad<=1.2;$rad+= $interval) {

        $buildcommand = "mkdir -p tmp_".$rad."_$speed;";
        $buildcommand = $buildcommand."cd tmp_".$rad."_$speed;";
        $buildcommand =
$buildcommand."../DiversitySimsO23D_13Mar18.R $rad $speed
$iterations;";
        $buildcommand = $buildcommand."cd ..;";
        $buildcommand = $buildcommand."ln -s
tmp_".$rad."_$speed/ModelOutput.csv
out_".$rad."_".$speed."_3d.csv;";

        runcommand
$dispatchbase.'\''.$moduleloads.$buildcommand.'\''."\n";


    }
}
```
